# HPA-axis function and grey matter volume reductions: imaging the diathesis-stress model in individuals at ultra-high risk of psychosis

**DOI:** 10.1038/tp.2016.68

**Published:** 2016-05-03

**Authors:** I Valli, N A Crossley, F Day, J Stone, S Tognin, V Mondelli, O Howes, L Valmaggia, C Pariante, P McGuire

**Affiliations:** 1 Department of Psychosis Studies, Institute of Psychiatry, Psychology and Neurosciences, King's College London, London, UK; 2 Institute for Biological and Medical Engineering, P. Catholic University of Chile, Santiago, Chile; 3 Department of Psychiatry, P. Catholic University of Chile, Santiago, Chile; 4 Department of Psychology, Institute of Psychiatry, Psychology and Neurosciences, King's College London, London, UK; 5 Department of Neuroimaging, Institute of Psychiatry, Psychology and Neurosciences, King's College London, London, UK; 6 Department of Psychological Medicine, Institute of Psychiatry, Psychology and Neurosciences, King's College London, London, UK; 7 National Institute for Health Research Mental Health Biomedical Research Centre at South London and Maudsley National Health Service Foundation Trust, King's College London, London, UK; 8 Institute of Clinical Science, Imperial College London, London, UK

## Abstract

The onset of psychosis is thought to involve interactions between environmental stressors and the brain, with cortisol as a putative mediator. We examined the relationship between the cortisol stress response and brain structure in subjects at ultra-high risk (UHR) for psychosis. Waking salivary cortisol was measured in 22 individuals at UHR for psychosis and 17 healthy controls. Grey matter volume was assessed using magnetic resonance imaging at 3 T. The relationship between the stress response and grey matter volume was investigated using voxel-based analyses. Our predictions of the topography of cortisol action as a structural brain modulator were informed by measures of brain glucocorticoid and mineralcorticoid receptor distribution obtained from the multimodal neuroanatomical and genetic Allen Brain Atlas. Across all subjects, reduced responsivity of the hypothalamus–pituitary–adrenal (HPA) axis was correlated with smaller grey matter volumes in the frontal, parietal and temporal cortex and in the hippocampus. This relationship was particularly marked in the UHR subjects in the right prefrontal, left parahippocampal/fusiform and parietal cortices. The subgroup that subsequently developed psychosis showed a significant blunting of HPA stress response, observed at trend level also in the whole UHR sample. Altered responses to stress in people at high risk of psychosis are related to reductions in grey matter volume in areas implicated in the vulnerability to psychotic disorders. These areas may represent the neural components of a stress vulnerability model.

## Introduction

The onset of psychosis is thought to involve interactions between psychosocial stressors in the environment and genetic factors that alter the brain such that there is an increased vulnerability to psychosis. The effects of environmental stressors on the brain are thought to be mediated by the hypothalamus–pituitary–adrenal (HPA) axis, which responds to stress by releasing cortisol into the bloodstream.^1^ Cortisol interacts with glucocorticoid (GRs) and mineralcorticoid (MRs) receptors which function as transcriptional regulators, but also modulate the responsiveness of the HPA axis via feedback inhibition of corticotropin-releasing hormone and adrenocorticotropic hormone release, such that homeostasis is re-established once stressors abate.^2^ The repeated or chronic exposure to stress leads to hyperactivity of the HPA axis, resulting in elevated basal cortisol levels and impaired responsiveness to further stress.

According to the neural diathesis-stress model of psychosis, the HPA axis mediates the relationship between exposure to stressors and the emergence of psychotic symptoms, with the suggestion that elevated cortisol levels augment dopamine synthesis.^3^ This model is supported by evidence that patients with a psychotic disorder have increased circulating levels of cortisol^4^ and a blunted cortisol response to stress,^5^ either in the form of experimental psychosocial stressors or the minor physiological stressor of awakening.^6^ The blunted cortisol response to stress is thought to reflect the impaired responsiveness of a desensitized system.^7^ Similar findings have recently been reported in individuals at ultra-high risk (UHR) of developing psychosis.^8, 9, 10, 11^

GRs and MRs are both expressed in the brain where corticosteroid hormones act as transcription factors and regulate gene expression.^1^ Data from animals and humans suggest that the HPA-axis stress-induced dysregulation and the consequent increased release of corticosteroids is associated with an enduring effect on brain structure, with the highest impact on areas undergoing developmental changes at the time of the insult.^2^ Thus, chronic corticosteroid exposure in rodents, both due to experimental administration or chronic stress, is associated with a reduction in dendritic branching in hippocampal and prefrontal regions.^12, 13^ Similarly, studies in humans exposed to stress or hypercortisolemia show reductions in hippocampal^14^ and prefrontal volume.^15^

There have been remarkably few studies of the relationship between alterations in HPA axis function and neuroimaging abnormalities in psychosis. An inverse correlation between hippocampal volume and cortisol levels has been observed in patients with first episode psychosis,^7^ although interpretation of this finding is complicated by the possible effects of illness or medication on both variables. These potentially confounding factors can be overcome by studying individuals at UHR for the disorder, who are usually medication-naive. UHR subjects show extensive alterations in grey matter volume irrespective of whether they subsequently develop the disorder,^16^ suggesting that these represent neural correlates of their vulnerability to psychosis. The only previous study in this group did not find a significant relationship between cortisol levels and either hippocampal or pituitary volume.^17^ However, the study used a regions of interest approach; therefore, the rest of the brain was not examined. Cortisol levels were assessed at a single time point via a blood sample.^17^ Serial samples provide a better index of HPA function, and the findings from blood samples can be confounded by the stress associated with venipuncture.

In the present study, we examined the relationship between the cortisol response on waking and whole-brain grey matter volume in UHR individuals. Cortisol was measured in serial salivary samples, and magnetic resonance imaging data were acquired on a 3 T scanner. We used the information on the regional expression of GR and MR in the brain^18^ to inform our predictions of the areas most likely to be related to HPA axis responsivity. On the basis of previous findings, we expected that UHR subjects would show a blunted waking cortisol response compared with controls. We then tested the hypothesis that there would be a significant relationship between blunted cortisol response and grey matter volume reductions in the hippocampus and the prefrontal cortex. A subsidiary hypothesis was that this relationship would be more pronounced in the UHR individuals than in controls.

## Materials and methods

### Ethical approval

The study was approved by the joint South London and Maudsley National Health Service Foundation Trust Ethics Committee and all participants gave written consent to participate after full details of the study were explained.

### Participants

Twenty-six individuals meeting criteria for an at-risk mental state (ARMS) were recruited from OASIS (Outreach and Support in South London),^19^ a clinical service for people at risk of developing psychosis within the South London and Maudsley National Health Service Foundation Trust. The diagnosis was based on Personal Assessment Crisis Evaluation criteria,^20^ as assessed by two expert clinicians using the comprehensive assessment of at-risk mental states (CAARMS)^21^ and confirmed at a consensus clinical meeting. All participants were antipsychotic naive at the time they took part in the study while five were taking antidepressant medication. Seventeen control subjects were recruited over the same period from the same sociodemographic area. Participants were aged 18 to 30 years and were excluded if their intelligence quotient was below 70, if they had a history of a neurological disorder or severe head injury or if they met DSM-IV criteria for an alcohol or substance dependence disorder other than nicotine. An additional exclusion criterion for control subjects was a family history of psychosis.

All the UHR participants were followed up by OASIS for at least 2 years after first contact and monitored for signs of transition to psychosis.

### Clinical measures

CAARMS,^21^ Positive and Negative Syndrome Scale (PANSS),^22^ Hamilton Anxiety Rating Scale (HAM-A)^23^ and Hamilton Depression Rating Scale (HAM-D)^24^ were used on the day of scanning to assess and rate symptom severity.

### Salivary cortisol

Salivary cortisol was collected in a naturalistic, non-clinical environment. Participants received verbal and written step-by-step instructions to use Salivettes (Sarstedt, Leicester, UK) and return them in a pre-paid envelope. Participants were instructed to wake up before 1000 h to collect saliva samples immediately at awakening (0  minutes) and then after 30 and 60 minutes. They were asked to abstain from consuming alcohol the night preceding collection and asked not to eat, drink, brush their teeth or engage in physical activity during the 60-minute collection period. Samples were stored at a temperature of −20 °C until they were centrifuged at 3500 rpm for 10  minutes at 6 °C to separate saliva from the pad. Saliva was then transferred from the Salivettes to microtubes and stored at −80 °C until a continuous, automated, competitive chemiluminescence immunoassay was performed using the Immulite immunoassay analyzer system (DPC; www.diagnostics.siemens.com)^25^ to determine free cortisol concentration. The percentage cross-reactivity of the antiserum with cortisone and prednisolone was 0.35% and 27.5%, respectively. The area under the curve for the cortisol awakening response (CAR) was calculated using cortisol levels at 0, 30 and 60  minutes after awakening with formulae described by Pruessner *et al.*^26^ The validity of the sampling is dependent upon timing, with delayed collection leading to an underestimation of peak response.^27^ A negative difference between the samples at time 0 and 30  minutes (Δ30) is considered indicative of delayed collection of the first sample with recommended exclusion from the analysis.^28^ Any participant with a missing sample or one characterized by very low salivary volume (<200 μl), one reportedly collected 15  minutes before or after the indicated time point or providing a negative Δ30 was therefore excluded from the study.

Demographic and cortisol measures were compared between the two groups using independent sample two-tailed *t*-tests, as variables were normally distributed. Chi-square was used for categorical variables.

### Image acquisition and analyses

Volumetric magnetic resonance images were acquired using a General Electric (Milwaukee, WI, USA) 3 T magnetic resonance system. A whole-brain three-dimensional coronal inversion recovery prepared spoiled gradient echo scan was acquired with echo time 2.82 ms, repetition time 6.96 ms, inversion time 450 ms and flip angle 20°.

Group-related differences in grey matter volume (GMV) were analysed using voxel-based morphometry, implemented in SPM8 software (http://www.fil.ion.ucl.ac.uk/spm) running under Matlab 7.4 (MatWorks, Natick, MA, USA). T1-weighted volumetric images were preprocessed using the Diffeomorphic Anatomical Registration Through Exponentiated Lie algebra (DARTEL)^29^ SPM8 toolbox, iteratively registering grey matter by nonlinear warping to a template generated using DARTEL to obtain a high-dimensional normalization.^29^ A homogeneity check across the sample was followed by smoothing with an 8-mm full-width at half maximum (FWHM) Gaussian kernel. The normalization protocol included a ‘modulatory step' to preserve information about the absolute grey matter values.^30^ We then looked for grey matter voxels in the normalized modulated smoothed data that correlated with CAR in all subjects. Age, gender and antidepressant medication were modelled in the analysis to reduce the potential impact of these variables on the findings. To identify specific changes not confounded by global volumetric differences, the proportional scaling option was used. We also looked for any existing differences in the relationship between cortisol response and cortical grey matter between UHR participants and controls. We thus used the general linear model to look for brain voxels in which this correlation differed according to the clinical status of the participants (UHR/control).

### Use of *a priori* biological information to guide statistical inferences

**Figure 1:**
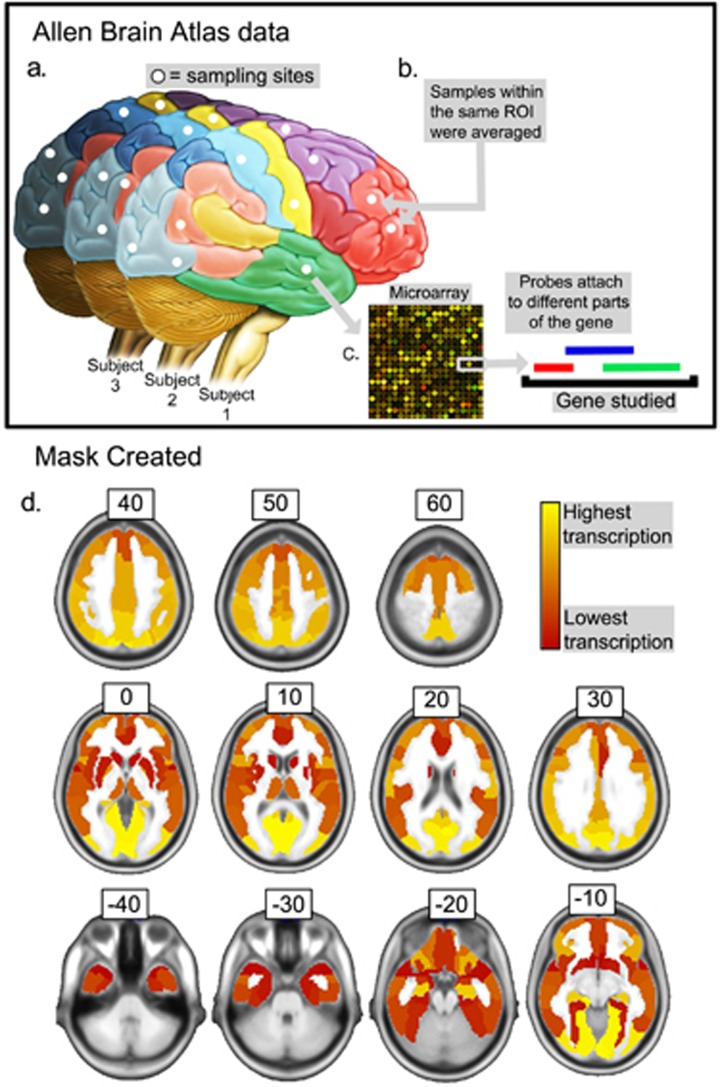
Method used to include *a priori* biological information. We used data from the Allen Brain Atlas (Allen Brain Atlas methodology summarized in (**a**–**c**) and described in detail in ref. 18) to create a new brain mask (**d**) used to flexibly threshold our results according to the expression of cortisol-binding receptors. (**a**) Information about expression levels of glucocorticoid (GR) and mineralocorticoid (MR) receptors was obtained from several parts of the brain of six healthy adults from the Allen Brain Atlas. (**b**) Samples obtained from the same region of interest of the template used were averaged. (**c**) Expression rates of probes targeting the same gene (MR or GR) were averaged. (**d**) Brain mask ranking regions according to their average expression of glucocorticoid and mineralocorticoid receptors in the healthy brain. The brain is shown in radiological convention (where the left side of the figure is the right side of the brain).

Neuroimaging studies usually involve the analysis of multiple univariate comparisons, posing a multiple-comparison problem. We here corrected our results based on the expression of corticoid receptors in the brain, using this to threshold our results. Our rationale was that regions that had high levels of these receptors were more likely to be influenced by cortisol, reducing the likelihood that a correlation with local grey matter volume would be a false positive. We used data from the Allen Brain Atlas,^18^ a multimodal atlas integrating neuroanatomical and gene expression information in humans. Briefly, the Atlas is based on tissue samples collected postmortem from anatomically diverse regions of six healthy adult human brains. Microarray analyses of the samples gave information on the RNA expression levels of a large number of genes for each of the regions sampled. The information on gene expression distribution across different regions of the brain was then used to build a whole-brain atlas. We retrieved the normalized transcription rates of the GRs and MRs on all the available sampled regions, then divided the brain into anatomically defined regions following a widely used template.^31^ Where one of the template regions included more than one sampling site, microarray information from the multiple samples was averaged. In addition, the Brain Allen project designed their microarray analysis such that more than one probe would target the expression of a specific gene. In this case, the expression levels of the GR and MR genes were inferred to be the average expression of the different probes targeting them. We took the mean across subjects, and on the basis that one-third of regulated genes are responsive to both receptor types,^32^ we averaged measures for both GR and MR expression in one *a priori* mask (Figure 1). Average transcription of the receptors across class and subjects were used to determine *a priori* probabilities of a false positive as described below.

We assumed that a biological relationship with cortisol levels would be most likely in brain areas where the expression of cortisol receptor genes was highest. In these areas, the statistical threshold was set at *P*<0.01, uncorrected for multiple comparisons. At the other extreme, in areas with the lowest expression of these genes, the statistical threshold was set at *P*<0.05, Bonferroni-corrected for all voxels within the mask. All areas were ranked according to their expression level and assigned a statistical threshold between *P*<0.01 uncorrected and *P*<0.05 Bonferroni-corrected. This range of probabilities was divided into equally sized steps, and a threshold was assigned to each region according to its rank.

## Results

### Demographic and clinical characteristics of the sample

26 individuals at UHR for psychosis and 17 healthy controls were originally included. Four UHR subjects had to be excluded due to the poor quality of the cortisol sampling, leaving 22 subjects with data for analysis.

Control and UHR individuals did not differ in terms of age (UHR mean [SD]=22.45 [4.08] years, controls mean [SD]=24.24 [4.21] years, df=37, *t*=−1.33, *P*=0.19) or gender (UHR females *n*=9, control females *n*=7, *P*=0.98). There was a trend for higher estimated premorbid intelligence in control participants (UHR mean [SD]=110.27 [10.46], controls mean [SD]=115.45 [7.04], df=37, *t*=−1.85, *P*=0.073).

As would be expected, UHR subjects had higher levels of psychopathology than controls as measured using the CAARMS and the PANSS and lower levels of functioning measured using the Global Assessment of Functioning (GAF). In addition, they showed higher levels of anxiety and depression symptoms as measured using the HAM-A and HAM-D (Supplementary Table 1).

The UHR participants were followed up for at least 2 years after the baseline assessments. Within that period, four subjects (18.2%) developed a psychotic disorder.

### Cortisol awakening response

**Figure 2:**
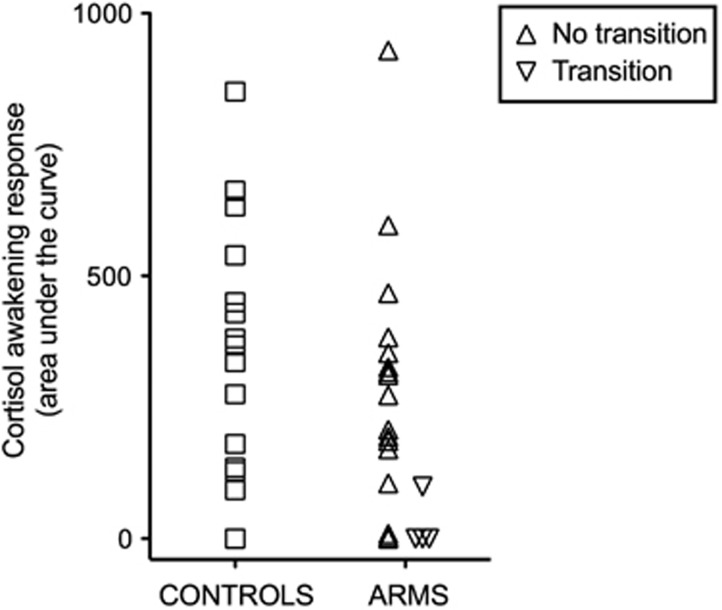
Cortisol awakening response (CAR) in ultra-high risk subjects and controls (nmol min/l). ARMS, at-risk mental state.

UHR participants showed lower levels of cortisol in response to awakening than controls, although this difference did not reach statistical significance (UHR mean [SD]=223.84 [233.52] nmol min/l, controls mean [SD]=320.97 [253.85] nmol min/l, df=37, *t*=−1.24, *P*=0.22). Visual inspection of the data (Figure 2) led to the identification of an outlier in the UHR group, confirmed by computing standard scores (*z*=3.02). After this subject was excluded, there was a strong trend for a between-group difference (UHR mean [SD]=190.29 [176.77] nmol min/l, controls mean [SD]=320.97 [253.85] nmol min/l, df=36, *t*=−1.87, *P*=0.07). The four subjects that subsequently transitioned to psychosis had CAR values significantly lower than controls (UHR-transition mean [SD]=24.75 [49.50] nmol min/l, controls mean [SD]=320.97 [253.85] nmol min/l, df=19, *t*=−2.281, *P*=0.034).

### Imaging

**Figure 3:**
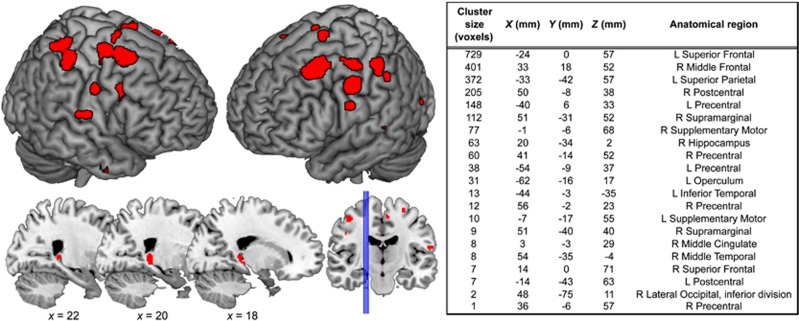
Brain regions showing a significant positive correlation with cortisol awakening response across all the subjects. L, left; R, right.

Across all subjects, there was a significant positive correlation between CAR and regional GMV in the superior frontal gyrus, the precentral and postcentral gyri and the supplementary motor cortex, bilaterally. Correlations were also evident in the right hippocampus, the right middle frontal, supramarginal, middle temporal and cingulate gyri, and in the left inferior temporal gyrus, superior parietal cortex and operculum (Figure 3). In all these regions, a blunted cortisol response was associated with smaller grey matter volume.

**Figure 4:**
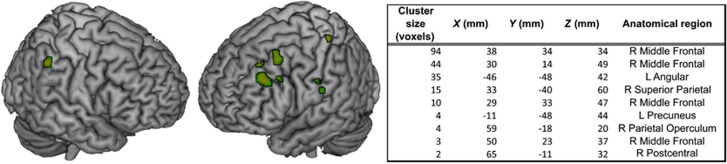
Brain regions where there was a stronger correlation between the grey matter volume and the cortisol awakening response in UHR subjects than in controls. L, left; R, right; UHR, ultra-high risk.

The correlation between CAR and GMV was significantly stronger in UHR individuals than in controls in the right middle frontal gyrus, the right superior parietal gyrus, the right parietal operculum, the right postcentral gyrus, the left angular gyrus, the left precuneus and the left parahippocampal/fusiform gyrus (Figure 4). Conversely, controls showed a stronger relationship in the left fusiform gyrus (Supplementary Figure 1).

## Discussion

This study examined the relationship between grey matter volume in individuals at UHR for psychosis^16^ and HPA axis abnormalities.^8^ Consistent with a previous finding in a larger sample,^8^ there was a trend for a blunting of the CAR in the UHR group. Our first major finding was that there was a significant positive relationship between CAR and regional GMV across all the subjects in bilateral frontal, parietal, and temporal cortices, and the right hippocampus, confirming our initial hypothesis. Consistent with our second hypothesis, this relationship was particularly marked in the UHR group, with impaired responsivity of the HPA axis linked to smaller GMV in the right prefrontal, left parahippocampal/fusiform and parietal cortices. The findings in the prefrontal and parahippocampal cortex are of particular interest, as these are the two brain regions most consistently implicated in animal models of psychosis,^33, 34^ neuroimaging studies of UHR subjects^16^ and patients with psychosis.^35^ The group differences at the neuroimaging level may have been more significant than those in the cortisol responses because they provide a more direct measure of the underlying pathophysiology.

Attenuated cortisol responses to stress in UHR individuals are thought to reflect a desensitization of the HPA axis that may increase the vulnerability to psychosis.^9^ It has also been suggested that HPA axis abnormalities may alter normal brain maturational processes. Exposure to stress during key periods of vulnerability may slow brain development^2^ as corticosteroids influence neurogenesis and neuroplasticity, affecting levels of neurotrophins such as BDNF.^36^ Elevated corticosteroid levels can also be neurotoxic, inducing regression of dendritic processes and decreasing neuronal survival following insults, thereby contributing to neuronal death.^37^ These effects could manifest as reductions in regional brain matter volume and could contribute to the emergence of psychotic symptoms.^38^

The hippocampus and prefrontal cortex have been found to be particularly susceptible to the effects of chronic or repeated exposure to stress in both animals and humans.^13, 36^ The hippocampus is most susceptible during the first years of life, when it is completing key maturational processes, while the prefrontal cortex remains vulnerable throughout the post-pubertal maturational period that coincides with the peak window of psychosis risk. Alterations in hippocampal volume may therefore be a correlate of early exposure to stress and may contribute to sensitization to stress due to the role of the hippocampus in the feedback control of the HPA axis.^5^ Desensitization of the HPA axis, mirrored by a heightened perception of daily experiences as stressful,^39, 40^ may contribute to further abnormalities through the effect of cortisol on brain regions undergoing neurodevelopment later in life, even in the absence of further trauma.

The mechanisms underlying stress sensitization are unknown, but changes in the dopaminergic circuitry have been suggested to play a role.^41^ Glucocorticoids augment dopamine activity, especially in the mesolimbic system^42^ and subjects at UHR for psychosis showed increased dopamine synthesis capacity^43^ and increased dopamine release in response to stress.^44^ Microdialysis investigations in rodents show that prefrontal GRs mediate the enhanced mesocortical dopamine efflux observed in response to acute stress, leading to an impairment of executive cognitive functioning.^45^ Impaired executive function is a key feature of the UHR state and of psychosis,^46^ and the frontal and parietal regions where we found correlations with the cortisol response mediate these processes. Our findings are thus in line with data from both animals and humans linking early social deprivation with structural and functional abnormalities in brain regions that mediate executive functions.^47, 48^ In patients with first episode psychosis, a blunted CAR predicts impaired executive functioning and poor treatment response.^49, 50^

The present study has a number of limitations. The sample size was modest, mainly because UHR subjects are difficult to recruit, and participation in the present study was relatively demanding, with subjects having to follow a complicated cortisol sampling protocol, undergo magnetic resonance imaging scanning and consent to long-term follow-up. A small sample size is a particular issue when studying UHR samples, which are characterized by significant clinical heterogeneity. Larger samples can be recruited through multicentre studies.^51^ The significantly attenuated CAR observed in subjects who subsequently progressed to an established psychotic illness needs therefore to be considered with caution due to the small number of UHR subjects who transitioned. A greater severity of anxiety and depression symptoms in the UHR participants also needs to be acknowledged, especially considering that a blunted CAR is not a finding specific to psychosis. Although all the UHR individuals were antipsychotic naive, four of them had been exposed to antidepressant treatment. We therefore covaried to minimize the effect of antidepressant use, which can alter cortisol levels.^52^ The correction we used for our imaging analysis was based on receptor density, but the range included *P*<0.01 uncorrected for regions where expression was highest. Finally, the information on receptor density distribution was derived from an atlas of the healthy adult brain, and we cannot exclude the possibility that corticosteroid receptor expression differs in UHR subjects because of epigenetic effects of stress.^53^ Strengths of the study include the naturalistic measurement of the CAR, minimizing the potentially confounding effects of measuring stress in an experimental setting, and the use of salivary as opposed to plasma sampling, which reduces the risk of stress being induced by the sampling procedure. We also informed our neuroimaging analysis with data on the central distribution of corticosteroid receptors.

To our knowledge, this is the first study to find a relationship between the cortisol response to stress and alterations in grey matter volume in people at high risk for psychosis. The data suggest that the neural diathesis-stress vulnerability model for psychosis may include the frontal, parietal and hippocampal areas. Their involvement is consistent with a wealth of data implicating these regions in the pathophysiology of psychosis.
