# Supplementary material for: HPA-axis function and grey matter volume reductions: imaging the diathesis-stress model in individuals at ultra-high risk of psychosis
Source: Transl Psychiatry. 2016 May 3;6(5):e797–. doi: 10.1038/tp.2016.68 (PMC5070043; doi:10.1038/tp.2016.68)
Supplement: Supplementary Information [file tp201668x1.doc]

**Supplementary Material**

Table S1 Clinical Measures

|  | UHR subjects mean (±SD) | Control subjects mean (±SD) | Significance  (p value) |
| --- | --- | --- | --- |
| CAARMS thought | 3.50 (1.30) | 0.12 (0.48) | <0.001 |
| CAARMS perception | 2.73 (1.61) | 0.35 (1.06) | <0.001 |
| CAARMS speech | 1.64 (1.84) | 0.29 (0.69) | 0.004 |
| PANSS positive | 12.18 (3.17) | 7.12 (0.33) | <0.001 |
| PANSS negative | 10.27 (3.34) | 7.06 (0.24) | <0.001 |
| PANSS general | 24.68 (4.33) | 16.65 (1.32) | <0.001 |
| PANSS total | 47.14 (8.66) | 30.82 (1.59) | <0.001 |
| GAF | 60.91 (12.65) | 85.94 (4.26) | <0.001 |
| HAM-A | 14.23 (10.62) | 1.41 (2.74) | <0.001 |
| HAM-D | 13.27 (8.18) | 0.94 (2.53) | <0.001 |

CAARMS: Comprehensive Assessment of At Risk Mental States, PANSS: Positive and Negative Symptoms Scale, GAF: Global Assessment of Functioning, HAM-A: Hamilton Anxiety Rating Scale, HAM-D: Hamilton Depression Rating Scale


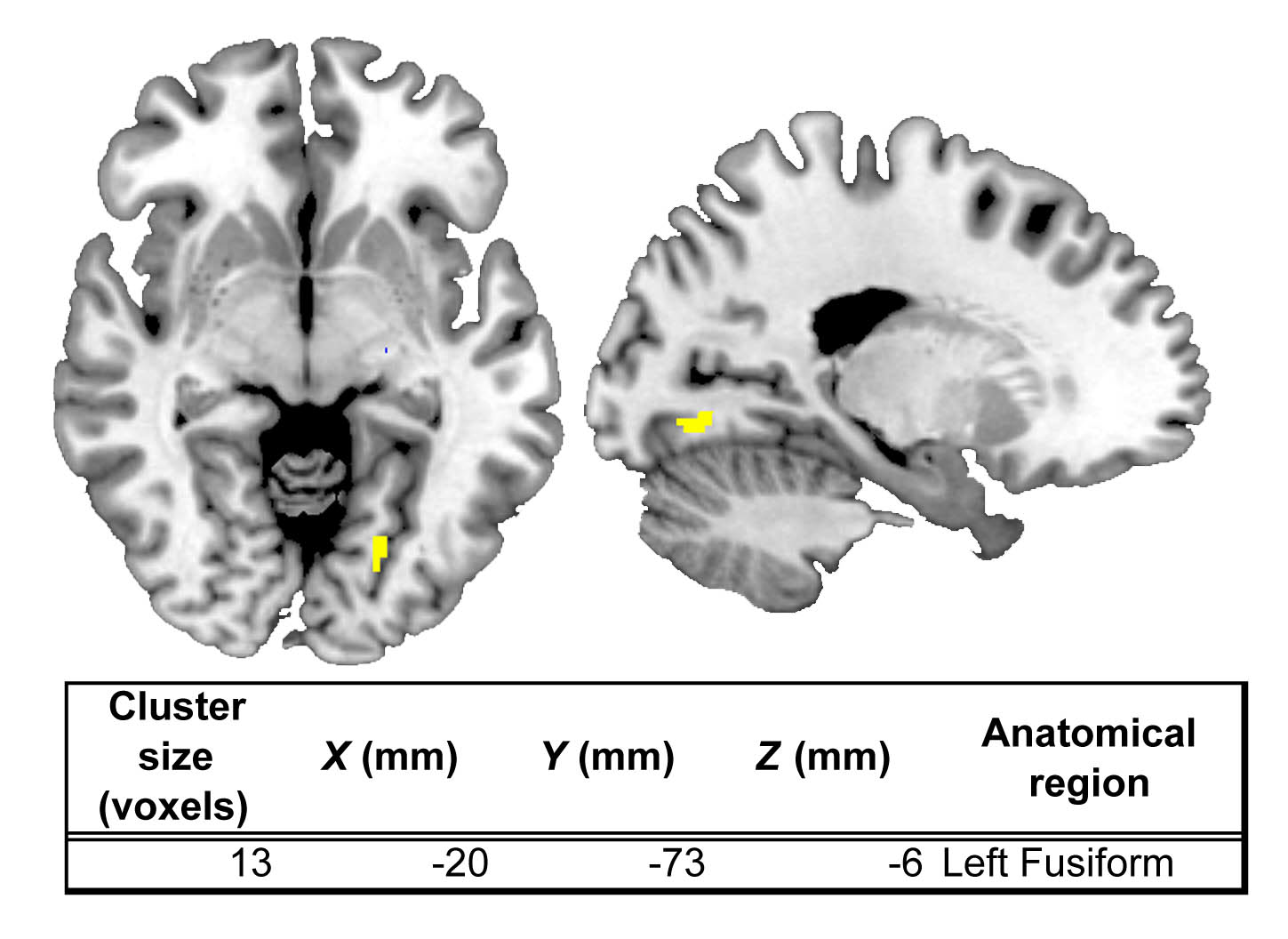


Figure S1 Brain regions where there was a stronger correlation between grey matter volume and cortisol awakening response in controls than in UHR individuals
